# Fusion of Cognitive Information: Evaluation and Evolution Method of Product Image Form

**DOI:** 10.1155/2021/5524093

**Published:** 2021-03-17

**Authors:** Shutao Zhang, Pengfei Su, Shifeng Liu

**Affiliations:** School of Design Art, Lanzhou University of Technology, Lanzhou 730050, China

## Abstract

In order to realize the stability and inheritance of image characteristics in the development process of a series of products, we comprehensively analyzed the cognitive differences among users, designers, and engineers and propose a multicriteria decision system for an intelligent design method of product forms based on a logistic regression model, relative entropy theory, and preference mapping (PREFMAP). First, from the perspective of the role characteristics of the design subjects, an equilibrium evaluation model was constructed using the logistic regression model and relative entropy theory. Second, combining the multidimensional perception space and the characteristics measurement of the product form, the fitness function of the image form was constructed based on PREFMAP. Third, a genetic algorithm was applied to establish the intelligent image-style-oriented design method, which could guide the image form development of a product series through innovative design. Lastly, the method was verified by taking Audi A4L series headlights as an example. And the image evaluation of the two new schemes was greater than that of the previous seven generations of headlights. The results verify the effectiveness and feasibility of the method. In this paper, we structured a relatively preliminary model to explain the fusion of cognitive information. More subjective and objective factors, algorithms, and image recognition technology need to be further studied to improve the model in our future work.

## 1. Introduction

Industrial design focuses on product factors such as function, shape, and information interaction, and it is a human-centered activity involving aesthetics, functions, and emotions. The meaning of design is not limited to creating things, but more importantly, giving things meaning and value. Users put forward higher requirements for the emotional design of products. As product demand has risen to emotional satisfaction, how to develop new products that meet users' emotional needs has become an important topic.

Product form innovation is the most direct and convenient way of product innovation, and it is an important method for enterprises to survive in fierce market competition [1]. Product form is the key point of product research and development. The external form of the product can directly affect a user's first impression of the product from many aspects, such as ergonomics, semantics, and aesthetics [2]. Product design is divided into structured and unstructured design expressions. The participating roles in product form evaluation are diverse, including users, designers, and engineers (“engineers” in the text refers to those who participate in design from a production perspective) [3]. Due to differences in the division of labor, cognition, design interpretation, and professional knowledge among design participants, cognitive conflicts in the design process inevitably exist [4].

The modern industrial design follows the human-centered design concept and emphasizes the importance of humanistic elements in the design process. This requires consideration of the cognition and feelings of users and engineers, encouraging them to participate in design decisions and collaborate with designers. User cognition is based on their own needs and aesthetic images. When evaluating products, they express themselves with qualitative language on the basis of their cognition. Designer cognition is based on their understanding of the design goals, and the design of the products is based on their own aesthetics. Engineer cognition is based on their own knowledge of structure, materials, and processing technologies to evaluate product forms from the perspective of structural design [5].

Product evaluation by the decision-making system should be comprehensive from users, designers, and engineers so that the product can meet multistandard perceptual needs and reduce image bias caused by cognitive differences between individuals and groups. Current studies on product form image evaluation mainly analyze the image perception differences of users, designers, and engineers from a macro perspective and rarely involve the driving factors behind the perception differences, especially the influencing factors of different cognitive subjects. Therefore, analyzing the cognitive differences of participating groups has become key to studying product form image evaluation.

In 1970, Professor Nagamachi proposed Kansei engineering [6]. As a product design method, Kansei engineering aims to transform user perceptions into design elements by quantifying people's perceptions of product forms. Its principle is to collect users' subjective evaluations of a set of products and then analyze and interpret the evaluation results to provide design rules or trend predictions through multiple statistical methods such as linear and nonlinear models, neural networks, and rough set theory [7]. In recent years, many scholars have applied Kansei engineering methods to study the cognition of users and designers. For example, by analyzing the cognitive difference between the two, Zhao found the balance of image cognition to improve the credibility of automobile appearance evaluation [3]. Later studies added engineers to the evaluation system to enhance the credibility of the decision-making system. For example, our research group introduced entropy theory to analyze differences in image cognition among users, designers, and engineers and then built a product form image evaluation model to guide the construction of typical product cases [8]. However, existing studies mainly analyze the image cognition differences of designers, users, and engineers from a macro perspective and give less consideration to the driving factors of cognitive differences.

According to the above analysis, we study the difference of cognitive subjects from the perspective of a dynamical system, evaluate the product form combined with a logistic regression model and entropy theory, and optimize the product form through a genetic algorithm. First, we analyzed the characteristics of each cognitive group with cognitive theory to determine product samples and target images. Second, the logistic regression model was used to analyze and quantify the cognitive differences of the three groups, and the entropy method and relative entropy were used to construct a comprehensive evaluation system on the basis of the dynamic analysis of cognitive differences. Lastly, we realized product form innovation through a genetic algorithm. While retaining the high-quality genes of high-scoring samples, the newly generated morphology was judged using the morphometric parameter constraint as the fitness function of the genetic algorithm to obtain the product form with a higher target image value.

The rest of this paper is organized as follows.  Section 2 presents the relationship between cognitive differences and cognitive motivation, and the feasibility of applying logistic regression, relative entropy, and genetic algorithm to establish a cognitive model. In Section 3, we propose the detailed process of constructing the cognitive model. In Section 4, we illustrate the research process of the model with the practical case of the Audi A4L series headlights.  Section 5 demonstrates the discussion of the model. Finally, Section 6 provides some brief conclusions.

## 2. Literature Review

### 2.1. Cognitive Differences and Cognitive Motivation

Cognitive psychology says that people's cognition of things is actually a process of information processing. Products stimulate the human sense organs with a series of symbolic language that is given by the designer. Users obtain the impression of products through the brain's information processing. Therefore, the process of product design is not only the designers shaping the product, but also the cognitive communication between designers, users, and engineers. In addition to the genetic and innate factors that affect cognitive subjects, also included differences in individuals' or groups' experience and memory and in their external environments [9]. Therefore, cognitive differences are actually the reason for the categorization of cognitive subjects.

In terms of research on the classification of cognitive subjects, Wang believed that the cognitive style of designers can be divided into impulsive and thinking [10]. In recent years, many scholars have studied cognitive science from the perspective of dynamical systems and proposed the dynamical systems theory of cognition. For example, taking interactivity and usability as the target driving force, Stepp established a human-computer interaction model by integrating the concept of emotion and cognition [11]. Jordan constructed a nonlinear cognitive dynamical system for recognition and classification with chaotic dynamics [12]. Zednik constructed dynamic equations to describe the mechanism of action between variables in the cognitive model [13].

Most research on cognitive dynamics focuses on cognitive science. In the field of product design, cognitive differences affect the development of the product form. This influence can be regarded as a kind of dynamic. Therefore, we introduced cognitive differences into the quantitative study of the product image form design and developed a cognitive dynamical system.

### 2.2. Logistic Regression

Logistic regression is a probabilistic nonlinear dynamic model. It was proposed by Malthus in the early study of the law of population growth and was summarized into mathematical equations by Wiherst [14]. As a multivariate analysis method, the logistic regression model is used to identify factors (*x*) that have a significant impact on a dependent variable (*y*) and predict the category of the dependent variable [15, 16]. It was successfully applied in disease prevention [17, 18], geology and meteorology [19, 20], and social problem research [21, 22].

In a design study, Li proposed a product image form design method based on ordinal logistic regression [23]. Combined with the logistic regression function, he established a mathematical model between the Kansei image and design elements and verified the feasibility of the method by taking office chairs as an example.

Due to its dynamic characteristics, the logistic regression model is often used to analyze influencing factors to predict outcome probability. Considering its good nonlinear regression characteristics, we applied a logistic regression model to explore the quantitative relationship between cognitive differences and product image evaluation on the basis of the survey data of cognitive subject characteristics.

### 2.3. Entropy Method and Relative Entropy

In 1856, the German physicist Rudolf Clausius proposed the concept of entropy to express the uniformity of energy distribution in space. In 1948, Claude Shannon introduced the concept of statistical entropy into information theory and expressed the uncertainty of information as an information measure on the basis of probability and statistical models. This is also called information entropy [24]. Shannon developed a specific expression for information entropy that makes information entropy more universal.

Driven by information entropy theory, entropy theory has more extensive applications in many fields such as the natural, social, and engineering sciences. Chen et al. introduced information entropy into the field of product form design. They developed a decision-making method for design order by calculating the information entropy of product style variables [25]. Qian et al. constructed a classification algorithm for artistic painting on the basis of information entropy and classified seven Eastern and Western artistic painting styles [26]. As an objective weighting method based on information entropy theory, the entropy method is used to calculate the weight of indicators through indicator information, and it is widely used in economic management and probability statistics [27–29].

In the field of design, Guo et al. evaluated mechanical product schemes by calculating the weight of each index attribute of the product with the entropy method [30]. In the previous research, we obtained the composite weight of the users, designers, and engineers for each target image through the entropy method and then calculated the composite evaluations of the samples [8]. The above studies were all based on the traditional linear superposition method, which has insufficient consideration of the balance of evaluation indicators and affects the objectivity of the evaluation result.

In the middle of the twentieth century, statisticians Kullback and Leibler proposed relative entropy to measure the similarity between two probability distributions. In recent years, relative entropy has been widely used in computer science [31], spectral information analysis [32], power system optimization [33], and evaluation method optimization [34–37].

In this research, we applied the entropy method to calculate the cognitive evaluation weights of users, designers, and engineers and introduced relative entropy equilibrium coefficients to modify the linear superposition evaluation mapping model to improve the objectivity and accuracy of evaluation.

### 2.4. Genetic Algorithm in Product Design

The genetic algorithm (GA) was first proposed by Professor John Holland in 1975. It is a computational model developed by simulating natural selection and the genetic mechanism in Darwin's theory of biological evolution. In product innovation design, designers face two problems: inheritance of the excellent genes of the product and innovation of the product form. The genetic algorithm provides effective tools to solve these problems. It can also improve design efficiency and shorten the product development cycle [38]. We conducted research in this regard [39, 40].

In genetic algorithms, as an important indicator of individual performance, the fitness function is the main basis for operation selection [41, 42]. Most existing applications of genetic algorithms in product design take manual evaluation or neural networks as the fitness function [43, 44]. However, the subjectivity of manual evaluation and the strict requirements of neural networks on data to a certain extent affect the objectivity and reliability of the fitness function. Therefore, in this study, we took product form and curve parameters as the fitness function to evaluate the characteristic measurements of product images. By introducing a cognitive space, the measurement range conditions of the target samples were developed to minimize the interference of human factors and improve the objectivity of the evaluation process. Petiot et al. verified the feasibility of this method with car headlights as a case study [45].

## 3. Methods

### 3.1. Research on Product Form Image Cognition

Image words and pictures related to the product are collected through the Internet, especially the official website of the product. Pictures are processed in grayscale with the same size and angle to exclude other image influence factors. The expert interview method is used to determine the target image vocabulary and select the target image words of the product.

The factors that affect the cognition of users, designers, and engineers are summarized from the relevant literature to construct semantic differential (SD) questionnaires suitable for the characteristics of the three types of cognitive subjects. Survey data were used as basic data for building the logistic regression model.

### 3.2. Cognitive Dynamic Model Based on Logistic Regression

The logistic regression model can predict dependent variables on the basis of multiple independent variables. The minimal required sample size is 10–20 times the number of independent variables, making the investigation less difficult.

Before constructing the logistic regression model, the usability of the survey data should be tested. The first step is to test the linear relationship between the continuous independent variable and the logit conversion value of the dependent variable using the Box–Tidwell method, which incorporates the interaction between the continuous independent variable and its natural logarithmic value into the regression equation for judgment. If the interaction item in the result is statistically significant (*P* > 0.05/*n*, where *n* is the total number of independent variables, interaction items, and intercept), this indicates a linear relationship between the continuous independent variable and the logit conversion value of the dependent variable. The next check can then be performed. If there is no linear relationship, the independent variable is converted into an ordinal categorical variable.

The second step is to test the multicollinearity between independent variables. Simple or multiple correlations between independent variables can lead to multicollinearity. We obtain the tolerance or variance inflation factor (VIF) through linear regression to judge the multicollinearity between independent variables. If the tolerance is less than 0.1 or the VIF is greater than 10, collinearity exists. One of the factors that lead to collinearity needs to be eliminated, but which factor is eliminated depends on the situation.

On the basis of the two tests, a logistic regression model can be constructed according to the survey data. Through data sampling, the regression coefficient of each cognitive influencing factor is calculated. The regression coefficients of various factors explain the change probability of image cognition and reveal the role and intensity of each influencing factor in promoting the change of image cognition. It can be organized as

(1) $$\begin{array}{c} p=\frac{\exp\left(\alpha +\beta_{1}x_{1}+\cdots +\beta_{n}x_{n}\right)}{1+\exp\left(\alpha +\beta_{1}x_{1}+\cdots +\beta_{n}x_{n}\right)}, \end{array}$$

where dependent variable *p* is the probability of image recognition; *x*_1_, *x*_2_, ..., *x*_*n*_ are independent variables; and *β*_1_, *β*_2_, ..., *β*_*n*_ are the logistic regression coefficients [8].

We selected several driving factors according to the significance level, applied the stepwise regression method to determine the main influencing variables, and analyzed the contribution of each influencing variable to image cognition on the basis of the regression coefficients. In this way, the probabilistic matrix of the image cognition of users, designers, and engineers was obtained. The probabilistic matrix of image cognition can be expressed as

(2) $$\begin{array}{c} X=\left[\begin{array}{ccc} X_{11} & \cdots & X_{1m}\\ \vdots & \ddots & \vdots \\ X_{n1} & \cdots & X_{nm} \end{array}\right], \end{array}$$

where *x*_*ij*_ represents the cognitive probability of the *j*-th cognitive subject of the *i*-th sample [30].

### 3.3. Computing Comprehensive Image Evaluation

The comprehensive image evaluation of serialized products should consider the cognitive differences of users, designers, and engineers. The entropy method is used to calculate the cognitive evaluation weights of the three, which are further corrected through relative entropy to obtain more objective and accurate product image evaluation.

In order to reduce the error of the evaluation, the image cognition probability matrix *X* is normalized to obtain decision matrix *X'* [46], which can be expressed as

(3) $$\begin{array}{c} X_{ij}^{\prime }=\frac{X_{ij}-\min\left\{X_{1j},\ldots ,X_{nj}\right\}}{\max\left\{X_{1j},\ldots ,X_{nj}\right\}-\min\left\{X_{1j},\ldots ,X_{nj}\right\}}. \end{array}$$

In order to reduce the influence of extreme value 0 on the evaluation result, when *x*_*ij*_′ = 0, it obtains a nonzero value of 0.001 to ensure the validity of the result. In addition, normalized data *x*_*ij*_′ are still denoted as *x*_*ij*_ for convenience [8]. The proportion of the *i*-th sample value of the *j*-th cognitive subject is *p*_*ij*_:

(4) $$\begin{array}{c} p_{ij}=\frac{X_{ij}}{\sum_{i=1}^{n}X_{ij}},\;i=1,\;2,\;\ldots ,\;n,\;j=1,2,\ldots ,m. \end{array}$$

The entropy value of the *j*-th cognitive subject can be calculated with the following equation [46]:

(5) $$\begin{array}{c} e_{j}=-k\sum_{i=1}^{m}p_{ij}\ln\left(p_{ij}\right),\;j=1,2,\ldots ,n, \end{array}$$

where *k* = 1/ln(*n*) > 0, *e*_*j*_ ≥ 0.

*W*_*j*_ is the weight of each cognitive subject's image cognition, which can be calculated with the following equation [46]:

(6) $$\begin{array}{c} w_{j}=\frac{1-e_{j}}{\sum_{j=1}^{n}\left(1-e_{j}\right)},\;j=1,2,\ldots ,n. \end{array}$$

Next, the image cognition weight vector *W* of users, designers, and engineers can be calculated.

From the extremum property of entropy, it can be seen that the closer the values of the components of the input vector are, the greater the entropy is. When the components are completely equal, maximal entropy is *S*_max_ = ln(*m*). Then, relative entropy is defined as

(7) $$\begin{array}{c} S_{x}=\frac{S_{\max}-S}{S_{\max}}, \end{array}$$

where *S*_*x*_ ∈ [0, 1]. It can be seen from the nature of entropy that the smaller the *S*_*x*_ is, the closer and more balanced the components of the input vector are [36, 37]. Therefore, it is appropriate to choose relative entropy *S*_*x*_ as the coefficient to quantify the equilibrium.

Before calculating relative entropy, the image cognition probability matrix *X* is standardized to obtain *p*_*ij*_′, which represents the proportion of the cognitive value of the *j*-th subject to the *i*-th sample; *p*_*ij*_′ can be expressed as

(8) $$\begin{array}{c} p_{ij}^{\prime }=\frac{x_{ij}}{\sum_{j=1}^{n}x_{ij}},\;i=1,2,\ldots ,m;\;j=1,2,\ldots ,n. \end{array}$$

Equation (9) allows for calculating the entropy value of the *i*-th sample:

(9) $$\begin{array}{c} S_{i}=-\sum_{j=1}^{n}p_{ij}^{\prime }\ln\left(p_{ij}^{\prime }\right),\;j=1,2,\ldots ,n. \end{array}$$

When the components are completely equal, maximal entropy is *S*_*i*max_ = ln(*n*) [46].

According to equation (7), the relative entropy of the *i*-th sample is calculated as

(10) $$\begin{array}{c} S_{xi}=\frac{S_{i\max}-S_{i}}{S_{i\max}},\;i=1,\ldots ,m. \end{array}$$

Taking relative entropy as the equilibrium coefficient, the linear evaluation model is modified to be an equilibrium evaluation model, which is expressed as

(11) $$\begin{array}{c} E_{i}=\left(1-S_{xi}\right)WX^{\prime }, \end{array}$$

where *E*_*i*_ is the comprehensive evaluation of the *i*-th sample, *X′* is the normalized image cognition probability matrix, *W* is the weight matrix of the cognitive subject, and *S*_*xi*_ is the relative entropy of the sample [8]. From this, we obtained a comprehensive evaluation of product image form based on the cognitive differences between users, designers, and engineers. According to the comprehensive evaluation value, good samples were selected as parent samples for evolution.

### 3.4. Product Form Evolution Based on GA

In the process of evolution, product forms are parameterized into a data matrix to perform genetic operations such as selection, crossover, and mutation:

1. Selection: according to the comprehensive image evaluation, the first two samples are selected as the parent samples, and the morphological contours of the samples are drawn and converted into a matrix of coordinates to prepare for crossover and mutation operations
2. Crossover: several coordinates are randomly selected from the coordinate matrix for interchange, and the number of points is controlled by cross-probability to realize the inheritance of excellent product form parameters
3. Mutation: coordinates are randomly selected from individuals generated by the crossover to change within a certain range, and the number of coordinates is controlled by mutation probability to achieve form innovation

The evolution system based on a genetic algorithm was implemented in MATLAB programming, and the optimization results are displayed through a human-computer interaction interface to prepare for manual selection and fitness function evaluation. Manual selection is mainly based on actual production and design experience to improve the feasibility of the product without affecting the objectivity of the fitness function.

In this paper, we take multiple measures of product form curve as the fitness function, so that the evaluation result could directly reflect the target image:

  Step 1: questionnaire survey. The subjects evaluated the different degrees of forms. The task of the subject was to evaluate the form difference between each pair of products in the range of 0 (exactly the same) to 10 (completely different); the result was output as difference matrix *Y*.
  Step 2: construction of perceptual space. The different degrees of forms were expressed in a low-dimensional perception space with multidimensional scaling (MDS) analysis. The principle of MDS is to find a set of points in a multidimensional space so that the distance between them corresponds to data in input difference matrix *Y* as much as possible. Its output is the perception space of product forms in the multidimensional space.
  Step 3: definition of morphological measures. By analyzing various objective physical characteristic measures of the product form, the role of characteristic measures in the perception process is to explain the dimensional relationship of the perception space. The measurement values (usually a linear relationship) related to the perception space dimensions are selected to define the perception dimensions.
  Step 4: image preference evaluation model. We propose to explain image evaluation with the perception dimension (fitting image evaluation to the perception space) through preference mapping (PREFMAP) analysis. PREFMAP has different processes to all existing models (vector model, circle, ellipse, and quadratic). Its purpose is to find the ideal area corresponding to the best value of image evaluation. We applied it to calculate the perception coordinates of the ideal area with the best value, which serves as the fitness function of the product form evolution design.

## 4. Empirical Study

**Figure 1:**
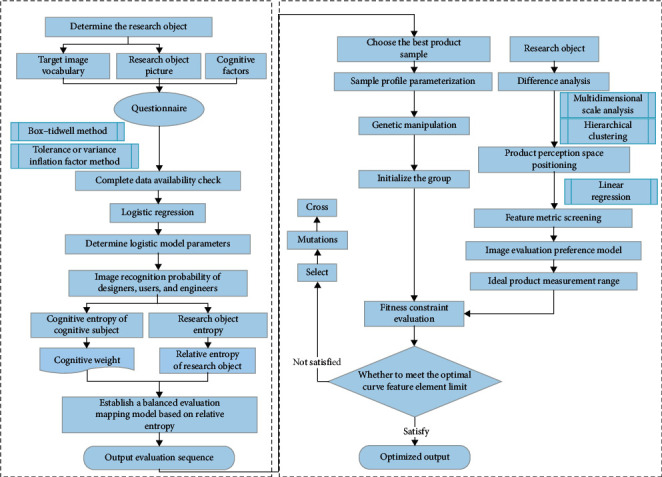
Flowchart.

A product form innovation method that systematically retains product image features is proposed; its implementation flow is demonstrated in Figure 1. The main mechanism of this method is to use logistic regression and relative entropy theory to evaluate the style image of existing serialized products from the perspective of the cognitive dynamic of users, designers, and engineers. Product samples with better evaluations were taken as the parent samples, and their image features were retained through the genetic algorithm, while product form innovation was carried out. Lastly, the form innovation results were judged by the fitness function based on PREFMAP to select innovative product forms that meet the development trend of product style serialization. The method is helpful in generating new product forms on the basis of classical stylized products with historic value.

### 4.1. Cognitive Analysis of Product Form Image

#### 4.1.1. Product Samples and Target Image Vocabulary

As the shining point of automotive design, headlight design has always been a hot field and has attracted much attention from design participants. Car headlights occupy a key position in perspective but also play a pivotal role in the image expression of the car design style. According to the literature [47], car headlights are the element that can best embody the image characteristics of the brand style, even exceeding the grille. Audi's headlights have always been recognized as a model of headlight design, and the intuitive and stable style image runs through the development of its series of car headlights. Therefore, we selected the headlights of the Audi A4L series as the research objects.

We collected and analyzed descriptions and photographs of Audi A4L series headlights through the official Audi website and professional auto websites such as https://www.audi.com/en.html, https://www.audi.cn/cn/web/zh.html and https://www.autohome.com.cn/lanzhou/. “Sporty” was selected as the target image word of the Audi A4L series headlights through the expert interview method from 42 image words. Twenty-four headlight pictures were processed into binary images through grayscale processing. Binary images were analyzed using the expert interview and KJ methods to obtain seven pictures representing the seven generations of car headlights of the Audi A4L series. The contours of the seven headlights were extracted with the same size and angle (see Table 1).

#### 4.1.2. Analysis of Cognitive Subject Characteristics

We analyzed the relevant literature on cognitive differences and obtained factors that affect user cognition, including gender, age, education level, temperament, and novelty of form. Factors that affect designer cognition include gender, age, education level, career experience, cognitive habits, design habits, and novelty of form. Factors that affect engineer cognition include gender, age, education level, career experience, processing technology, and novelty of form.

#### 4.1.3. Questionnaire Design and Survey Results

**Figure 2:**
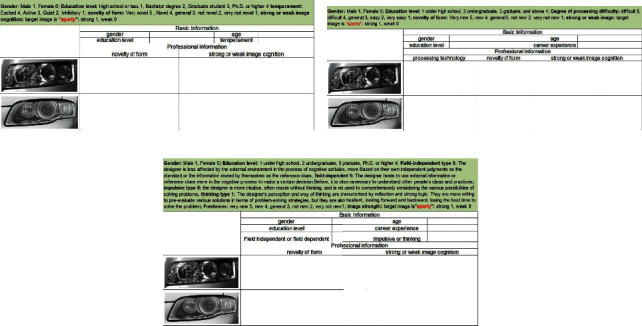
Questionnaires (a) for users, (b) designers, and (c) engineers.

In the questionnaire, the subjects directly filled numbers in the terms of age and career experience. Gender, cognitive habits, and design habits had 0 and 1 as options. The remaining projects were designed in the form of a Likert scale. Taking education level as an example, 1 meant high school, 2 meant undergraduate, 3 meant master, and 4 meant doctorate. In addition, in order to further explore the characteristics of each cognitive subject, a survey of the cognitive strength of the target image was added to the questionnaire, with 1 indicating strength and 0 indicating weakness. The questionnaires are shown in Figure 2.

We distributed 110 questionnaires to users, designers, and engineers, respectively. Returned valid questionnaires were 102 user questionnaires, 105 designer questionnaires, and 100 engineer questionnaires. The collected data were classified into the basic data according to the headlight generation for cognitive difference analysis. The survey data of 20 users of the first-generation headlights are shown in Table 2.

### 4.2. Cognitive Dynamic Analysis Based on Logistic Regression

The Box–Tidwell method was used to test the linear relationship between the continuous independent variables and the logit conversion values of the dependent variables. Taking user survey data of the first-generation headlights as an example, the test results are shown in Table 3.

There were six variables and one constant. “Age” was a continuous independent variable. “Gender,” “education,” “temperament,” and “experience” were categorical independent variables. “Ln_age by age” was an interaction term. We chose significance level *T* = 0.007 (that is, 0.05/7). According to this significance level, the *P* value of the interaction term in this study was higher than 0.007, so there was a linear relationship between all continuous independent variables and the logit conversion values of the dependent variables. All data passed the test.

Tolerance and the VIF were calculated through linear regression to evaluate the multicollinearity between independent variables. Taking the user survey data of the first-generation headlights as an example, the test results are shown in Table 4.

Results showed that the tolerances of all items were greater than 0.1, and VIFs were less than 10. Both test results showed that the data were valid and could be used to construct a logistic regression model. The results of logistic regression are shown in Table 5.

According to the logistic regression results in Table 5, the significance levels of the two variables of education and temperament were less than 0.05, indicating that these two variables had a significant impact on the “sporty” image. In logistic regression, the Exp (*β*) value of the independent variable being 1 means that the independent variable has no effect on the dependent variable. Exp (*β*) 95% confidence interval refers to calculating an interval according to predetermined probability so that it can contain the unknown overall mean. According to the results, the 95% confidence interval of Exp (*β*) for gender was 0.803∼5.572, for age was 0.916∼1.063, and for usage experience was 0.700∼2.533. The 95% confidence interval of Exp (*β*) of the three independent variables contained 1, which shows that the value of Exp (*β*) may have been 1, and there was no statistical significance in the correlation with the “sporty” image. Therefore, the three were invalid variables and not included in the regression model. Lastly, bringing the regression coefficient (*ß*) and the mean value of the effective variables into equation (1), the user cognition probability of the “sporty” image of the first-generation headlights was calculated to be 0.622.

By repeating the above steps, we obtained the perception probability of the “sporty” image of Audi A4L series headlights by users, designers, and engineers, as shown in Table 6. The data constituted image cognition probability matrix *X*.

According to equation (3), matrix *X* was normalized, and the calculation result formed normalized image cognition probability matrix *X′*, as shown in Table 7.

The normalized data in Table 7 were brought into equation (4) to obtain the cognitive proportions of the samples, as shown in Table 8.

Data in Table 8 were brought into equation (5) to obtain the entropy values of the cognitive subjects. Entropy values were brought into equation (6) to obtain the weights of cognitive subjects that formed weight matrix *W*, as shown in Table 9.

Image cognition probability matrix *X* was longitudinally normalized through equation (8) to obtain the proportion of cognitive subjects to each sample, as shown in Table 10.

The proportion in Table 10 was processed by equation (9) to obtain the entropy value of each sample. When the proportion of the cognitive probability of subjects was completely equal, maximal entropy was S_*imax*_ = ln(3), and the relative entropy of each sample was calculated according to equation (10). With normalized matrix *X′*, weight matrix *W*, and relative entropy, we obtained the comprehensive evaluations of the “sporty” image of Audi A4L series car headlights through equation (11), as shown in Table 11.

### 4.3. Construction of Fitness Function

#### 4.3.1. Perception Space of Product Form

**Figure 3:**
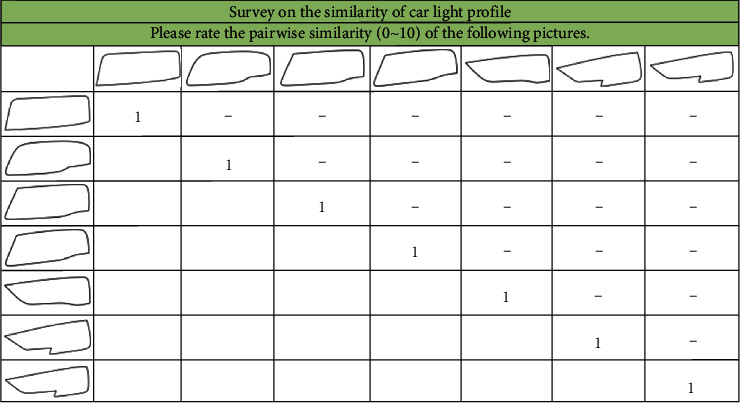
SD questionnaire of samples.

To study the product form difference of Audi A4L series car headlights, an SD questionnaire was designed, as shown in Figure 3. We distributed and collected 10 valid questionnaires and calculated the average to obtain the difference matrix, as shown in Table 12.

**Figure 4:**
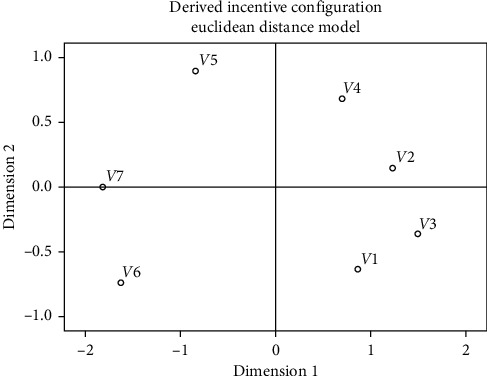
Perception space of product form.

The difference matrix was processed through MDS to construct a two-dimensional perception space of the product form, as shown in Figure 4. The coordinates of the product form in the perception space are shown in Table 13.

**Figure 5:**
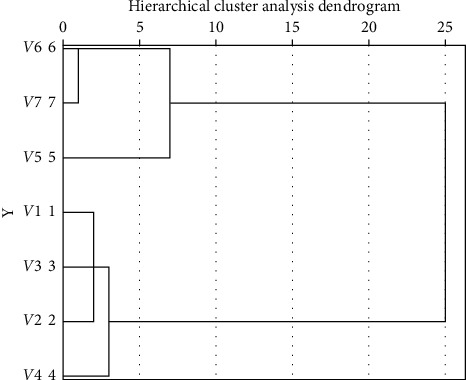
Hierarchical clustering result of the difference matrix.

**Figure 6:**
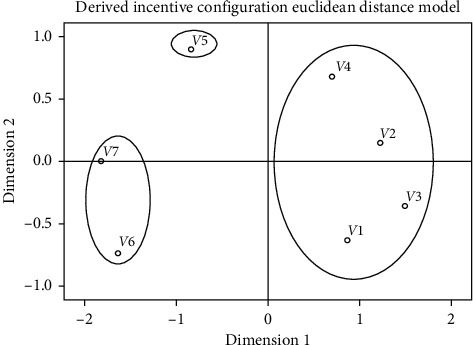
Grouping in product perception space.

Since it is impossible to see the classification of the product forms from Figure 4 and Table 13, we introduced hierarchical clustering to analyze the difference matrix, and the results are shown in Figure 5. According to the results, car headlights can be divided into three groups: (1) composed of the first-, second-, third-, and fourth-generation headlights; (2) only composed of fifth-generation headlights; and (3) composed of sixth- and seventh-generation headlights. The grouping in the product perception space is shown in Figure 6.

#### 4.3.2. Product Form Image Evaluation Model and Fitness Function

According to the grouping of headlight forms in the perception space, we speculated that the area of the headlights could explain the second dimension of the perception space: the first group of headlights on the right has a larger area, and the third group of headlights on the left has a smaller area. In addition, as another influencing factor of the form difference, the lines and angle of the headlights also play a role in the positioning of the perception space, but the specific mechanism cannot be explained at the intuitive level. Therefore, it was necessary to carry out specific measurements on the form characteristic measures of the headlights and explore the relationship between characteristic measures and the first dimension.

**Figure 7:**
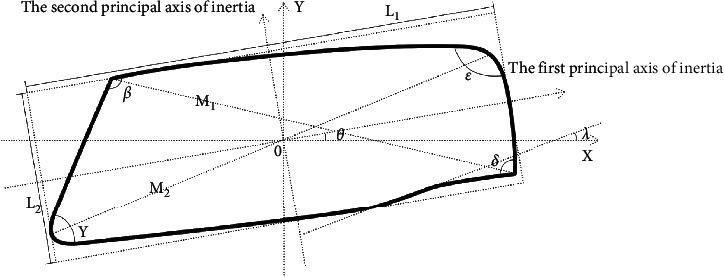
Characteristic measures of fourth-generation headlights.

Rhinoceros was applied to trace the shape of the headlights. The form characteristic measures included area (*S*), length (*L*1), width (*L*2), Diagonal 1 (*M*1), Diagonal 2 (*M*2), the first principal moments of inertia (PMOI) of the center of gravity (*I*1), the second PMOI of the center of gravity (*I*2), Angle 1 (*ε*), Angle 2 (*ß*), Angle 3 (*γ*), Angle 4 (*δ*), the angle between first principal axis of inertia and horizontal axis (*θ*), the angle between the bottom line and *X*-axis (*λ*), the slope of the bottom line to the first inertial principal axis (*K*), aspect ratio (*L*1/*L*2), diagonal ratio (*M*1/*M*2), and the ratio of the PMOI of the center of gravity (*I*1/*I*2). The processing results of the fourth-generation headlights are shown in Figure 7.

The measurement results of all headlights are shown in Table 14.

The measurement results show that the area of the headlights can explain the second dimension of the perception space from the perspective of a linear relationship. For other form characteristic measures, we constructed a linear regression model between the form characteristic measures and the first-dimension coordinate values. The following indices were used to verify the quality of the linear model to select form characteristic measures. The first is the model's goodness of fit *R*2. The larger that value is, the better the linear goodness of fit is. The second one is the mean absolute deviation (MAD), which represents the prediction accuracy of the model. The smaller the MAD is, the better the prediction accuracy is. The scatterplot was used to judge the linear characteristics of the model.

The results of the linear regression index are shown in Table 15

According to data in the table, the form characteristic measures with a larger *R*2 were *L*2, *ε*, *K*, and *L*1/*L*2. The form characteristic measures with a smaller MAD were *L*2, *δ*, *K*, and *L*1/*L*2. The three form characteristic measures of *L*2, *K*, and *L*1/*L*2 had good reliability for the linear regression model. Therefore, *L*2, *K*, and *L*1/*L*2 were chosen to explain the perceptual coordinate positioning in the first dimension. In summary, *S*, *L*2, *K*, and *L*1/*L*2 were taken as the consideration objects in the fitness function of the product form.

**Figure 8:**
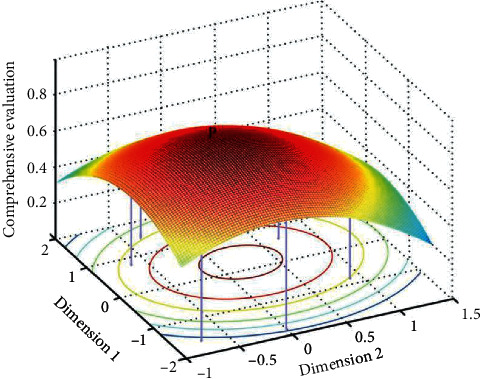
Image evaluation space.

The comprehensive image evaluations of the headlights were introduced into the two-dimensional product form perception space as the third dimension, and an image evaluation space (parabola) with ideal points was defined by the PREFMAP, as shown in Figure 8. Its expression is given by

(12) $$\begin{array}{c} P\left(D_{1},D_{2}\right)=a\left(D_{1}^{2}+D_{2}^{2}\right)+bD_{1}+cD_{2}. \end{array}$$

**Figure 9:**
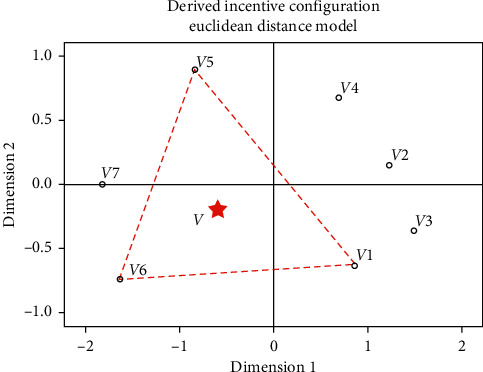
Ideal range of image evaluation in perception space which is located in the red triangle area.

The space model is a paraboloid with vertices. If *a* > 0, the optimal solution was located at the lowest point of the model. If *a* < 0, the optimal solution was located at the ideal highest point of the model. According to Figure 8, point *P* was the ideal highest point of image evaluation, and its mapping point *V* in the two-dimensional perception space was located in the triangular area surrounded by the first-, fifth-, and sixth-generation products with high image evaluation, as shown in Figure 9. According to parameters *S*, *L*2, *K*, and *L*1/*L*2 of the three products, the parameter ranges of the ideal form measurement of the target product were obtained, as shown in Table 16.

**Figure 10:**
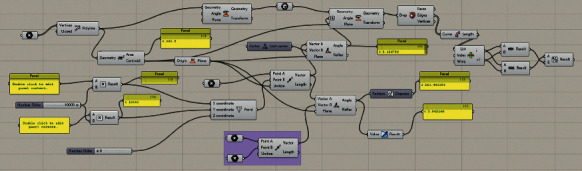
Interactive measurement parameter detection system based on Grasshopper.

Data processing of an ideal measurement is programmed through the parametric design function of Grasshopper software to establish an interactive measurement parameter detection system (IMPDS), as shown in Figure 10. By inputting the coordinates of the characteristic points of the form to be tested, the values of *S*, *L*2, *K*, and *L*1/*L*2 can be output, and the fitness function of the product form is lastly established.

### 4.4. Product Form Evolution

We took samples 1 and 5 with higher image values as the parent samples and, respectively, extracted 18 characteristic points on their outline through the Adobe Illustrator software. Taking the center of the form as the origin, a coordinate system was established to measure the horizontal and vertical coordinates of the 18 points, as shown in Table 17.

**Figure 11:**
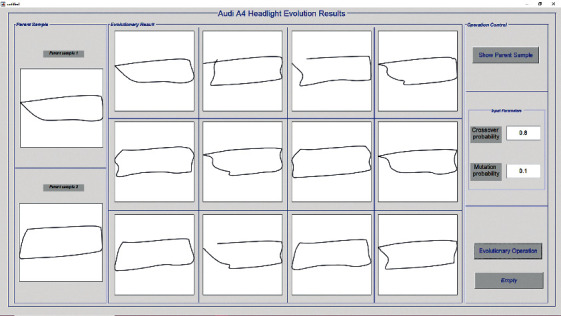
Product form evolution design system.

The operation process was implemented in the form of MATLAB programming, and the optimization results were displayed in a human-computer interaction interface, as shown in Figure 11.

By changing the probability of crossover and mutation, the evolution system was continuously operated to obtain a large number of new product forms. Through manual evaluation, we selected better forms whose coordinates were input into the IMPDS to be evaluated. Some evaluation results are shown in Table 18.

**Figure 12:**
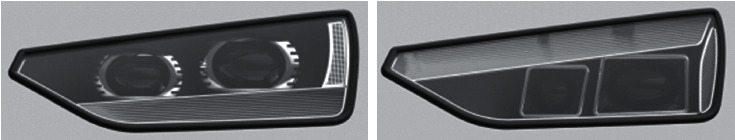
New schemes.

According to the ideal measurement range, we chose the fifth form in Table 18 as the optimized sample. Next, we designed the product details through 3D software and obtained two schemes [48], as shown in Figure 12.

The two schemes were combined with the “sporty” image to establish a seven-level SD questionnaire. The object of this survey was an expert group composed of 15 users, designers, and engineers, and 15 valid questionnaires were collected. Statistical analysis of the results of this questionnaire is shown in Table 19.

Results showed that the image scores of the two schemes were greater than the comprehensive image evaluation results of the previous seven generations of headlights. The optimized design method that we proposed was feasible and can provide a reference for designers to carry out product serialization design.

## 5. Discussion

The cognitive differences of subjects are driven by their own factors, but it is difficult for designers to consider the perceptual needs of each cognitive subject in the design process. As a multivariate analysis method, logistic regression is characterized by its ability to predict results by analyzing influencing factors. We use the characteristics of logistic regression to effectively promote the integration of cognitive differences between different subjects and promote the emotional unity of cognitive subjects.

In this research, genetic algorithm and fitness evaluation mechanism mainly play the role of assisting designers to develop innovative schemes with target images. The design process can be carried out in a computer environment, which is conducive to the realization of intelligent design. It can reduce the designer's work burden to a certain extent and realize the retention of the target image in schemes.

The recognition subject of product images is humans. As a complex process, human cognition is affected by a variety of objective factors, such as education, religion, technology, and culture. This study only considered some representative subjective factors, which may have an impact on the results of the study. In addition, the quadratic parabolic model cannot completely simulate product image evaluation in the perception space. In this case, it is difficult to accurately locate the ideal point.

Cognition is affected by various factors, including not only product form, but also CMF (Color, Material & Finishing), use environment, and other factors, which lead to the uncertainty of data. In our next work, the construction of a perception space should be based on different products to expand into a multidimensional perception space according to elements such as product form, material, and color [49, 50]. And the establishment of the linear relationship must consider multiple dimensions to select the appropriate measurement, and the shape of the ideal area also needs to consider the number of dimensions.

As mentioned in the previous paragraph, in order to obtain a better model accuracy, we intentionally ignore the product color, material, finishing, and other factors. In order to weaken the influence of CMF, we had a unified grayscale processing for all product pictures. Compared with color pictures, gray pictures have a different impact on users' cognition. This also provides us with a new research idea in image cognition. Next, we will make a cognitive comparative study of color pictures and black and white pictures with preimage processing [51, 52].

In this study, we introduced a basic formula of entropy theory to explain the cognitive balance and established a primary model to prove the effectiveness of the method. To further improve the accuracy of the model, we will focus on the exploitation of a new generation of computing approaches based on neuro-fuzzy approaches and fuzzy entropy principle [53, 54].

Lastly, the overall design of car headlights needs to consider the form and layout of internal elements. How to use systematic methods to rationally integrate the external form and internal elements of the headlights to highlight the target image is the focus of future research. We carried out research in this area and achieved some results [48].

## 6. Conclusions

In this paper, we proposed a multicriteria decision method of product form images based on a logistic regression model. First, multidimensional perception space was defined according to the evaluation results. Second, through the linear relationship between product forms and images, some characteristic measures were selected as the fitness function to judge the optimization result of the genetic algorithm. Lastly, a computer-aided design system was constructed; results showed that this method could guide product forms toward the target image and that it had better performance for improving product image design.

Although car headlight design was used as a case in this paper, the method can be applied to other product form designs. With the development of science and technology, product image design pays increasing attention to objective factors such as art, culture, and lifestyle to enhance its integrity and feasibility.

## Figures and Tables

**Table 1:** Headlights of Audi A4L series.

Sample	Year	Binary image	Contour
1	2001	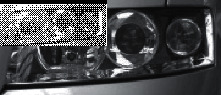	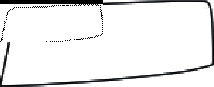
2	2006	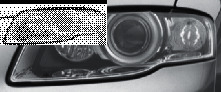	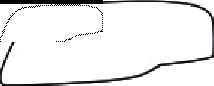
3	2009	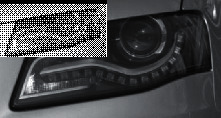	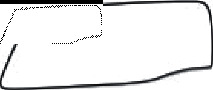
4	2010	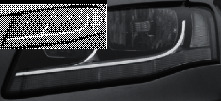	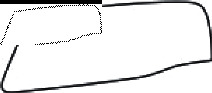
5	2013	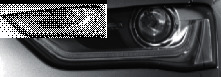	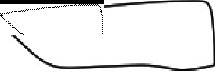
6	2017	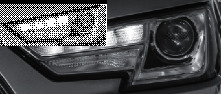	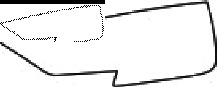
7	2018	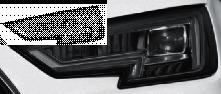	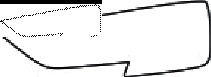

**Table 2:** Survey data of 20 users of first-generation headlights.

User	Gender	Age	Education	Temperament	Novelty	Cognitive strength
1	1	22	2	2	2	2
2	1	26	3	3	2	2
3	2	24	2	3	2	2
4	1	25	3	2	1	1
5	2	24	2	2	2	2
6	2	24	3	3	2	2
7	2	24	2	2	2	1
8	2	24	2	3	3	1
9	1	24	2	3	2	1
10	2	51	1	2	1	2
11	1	30	3	2	1	1
12	2	26	3	3	2	1
13	1	25	3	3	2	1
14	2	26	4	2	1	1
15	1	29	2	2	1	2
16	1	26	3	2	1	1
17	1	18	2	1	3	1
18	2	24	3	3	1	1
19	2	25	3	4	3	2
20	2	24	2	2	2	1

**Table 3:** Test results of first-generation headlights with the Box–Tidwell method.

	Variable	*β*	SE	Wald's	df	Sig.	Exp (*β*)
Step 1	Gender	0.765	0.499	2.349	1	0.125	2.148
	Age	–0.343	1.359	0.064	1	0.801	0.710
	Education	–0.883	0.478	3.421	1	0.064	0.413
	Temperament	1.171	0.405	8.371	1	0.004	3.226
	Experience	0.278	0.330	0.711	1	0.399	1.321
	Ln_age by age	0.073	0.299	0.059	1	0.808	1.075
	Constant	–0.266	9.601	0.001	1	0.978	0.766

**Table 4:** Multicollinearity test between independent variables.

Model	Collinearity statistics	
	Tolerance	VIF
Gender	0.863	1.159
Age	0.844	1.185
Education	0.754	1.326
Temperament	0.778	1.285
Experience	0.840	1.190

VIF, variance inflation factor.

**Table 5:** Results of logistic regression.

	*β*	SE	Wald's	df	Sig.	Exp (*β*)	95% CI of Exp (*β*)	
							Minimum	Maximum
Gender	0.749	0.494	2.300	1	0.129	2.116	0.803	5.572
Age	–0.013	0.038	0.123	1	0.726	0.987	0.916	1.063
Education	–0.918	0.458	4.012	1	0.045	0.399	0.163	0.980
Temperament	1.166	0.406	8.267	1	0.004	3.209	1.449	7.105
Experience	0.286	0.328	0.762	1	0.383	1.332	0.700	2.533
Constant	–2.544	2.127	1.430	1	0.232	0.079		

**Table 6:** Image cognition probability matrix *X*.

Subject	Sample 1	Sample 2	Sample 3	Sample 4	Sample 5	Sample 6	Sample 7
Users	0.622	0.819	0.132	0.286	0.418	0.885	0.342
Designers	0.420	0.023	0.671	0.653	0.724	0.165	0.915
Engineers	0.634	0.658	0.808	0.474	0.512	0.713	0.386

**Table 7:** Normalized image cognition probability matrix.

Subject	Sample 1	Sample 2	Sample 3	Sample 4	Sample 5	Sample 6	Sample 7
Users	0.651	0.912	0.001	0.205	0.380	1	0.279
Designers	0.445	0.001	0.726	0.706	0.786	0.159	1
Engineers	0.588	0.645	1	0.209	0.299	0.775	0.001

**Table 8:** Cognitive proportions of samples.

Subject	Sample 1	Sample 2	Sample 3	Sample 4	Sample 5	Sample 6	Sample 7
Users	0.190	0.266	0.001	0.060	0.111	0.292	0.081
Designers	0.116	0.001	0.190	0.185	0.206	0.042	0.262
Engineers	0.167	0.183	0.284	0.059	0.085	0.220	0.001

**Table 9:** Entropy values and weights of cognitive subjects.

Item	Users	Designers	Engineers
Entropy value	0.848	0.871	0.865
Weight	0.365	0.310	0.325

**Table 10:** Proportion of cognitive subjects.

Subject	Sample 1	Sample 2	Sample 3	Sample 4	Sample 5	Sample 6	Sample 7
Users	0.371	0.546	0.082	0.202	0.253	0.502	0.208
Designers	0.251	0.015	0.417	0.462	0.438	0.094	0.557
Engineers	0.378	0.439	0.501	0.336	0.309	0.404	0.235

**Table 11:** Relative entropy and comprehensive image evaluations of samples.

Item	Sample 1	Sample 2	Sample 3	Sample 4	Sample 5	Sample 6	Sample 7
Relative entropy	**0.015**	0.313	0.166	0.048	**0.024**	0.149	0.096
Comprehensive evaluation	**0.555**	0.357	0.433	0.439	**0.530**	0.515	0.483

**Table 12:** Difference matrix of samples.

	Sample 1	Sample 2	Sample 3	Sample 4	Sample 5	Sample 6	Sample 7
Sample 1	0	3	2	4	6	7	8
Sample 2	3	0	3	3	6	8	9
Sample 3	2	3	0	2	8	9	9
Sample 4	4	3	2	0	4	8	7
Sample 5	6	6	8	4	0	5	4
Sample 6	7	8	9	8	5	0	2
Sample 7	8	9	9	7	4	2	0

**Table 13:** Coordinates of product form in perception space.

	*V*1	*V*2	*V*3	*V*4	*V*5	*V*6	*V*7
*X* (dimension 2)	0.8623	1.2294	1.4933	0.6994	–0.8379	–1.6281	–1.8184
*Y* (dimension 1)	–0.6323	0.1482	–0.3596	0.6825	0.8977	–0.7375	0.0011

**Table 14:** Characteristic measurement results.

Measure	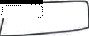	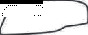	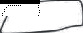	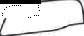	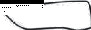	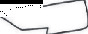	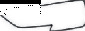
*S*	801.53	818.07	836.07	779.71	643.79	641.87	567.79
*L*1	50.41	52.87	51.42	50.85	51.08	51.29	49.74
*L*2	18.14	19.35	20.02	18.43	16.22	18.28	16.45
*M*1	46.45	44.35	44.55	44.40	52.20	51.47	49.49
*M*2	51.95	52.49	52.06	50.85	39.05	40.16	40.30
*I*1	147888.17	152134.51	150447.91	137237.04	111607.23	104426.57	91719.38
*I*2	18966.51	19501.91	21778.34	18104.38	10760.69	11818.92	8427.82
*ε*	116.50	96.44	103.25	101.07	98.69	104.55	119.19
*β*	128.66	140.65	119.63	125.86	53.96	46.41	49.80
*γ*	76.07	93.19	78.17	70.60	136.94	142.80	143.83
*δ*	106.58	111.46	100.18	94.73	104.74	91.06	100.12
*θ*	5.37	8.00	7.37	9.67	0.44	3.63	3.40
*λ*	0	30.92	16.08	15.51	–14.61	–110.37	–114.53
*K*	0.01	0.50	0.21	0.23	–0.21	2.78	2.40
*L*1/*L*2	2.78	2.73	2.57	2.76	3.15	2.81	3.02
*M*1/*M*2	0.89	0.85	0.86	0.87	1.34	1.28	1.23
*I*1/*I*2	7.80	7.80	6.91	7.58	10.37	8.84	10.88

S, area; *L*1, length; *L*2, width; *M*1, Diagonal 1; *M*2, Diagonal 2; *I*1, first principal moments of inertia (PMOI) of the center of gravity; *I*2, second PMOI of the center of gravity; *ε*, Angle 1; *β*, Angle 2; *γ*, Angle 3; *δ*, Angle 4; *θ*, the angle between first principal axis of inertia and horizontal axis; *λ*, the angle between the bottom line and *X*-axis; *K*, the slope of the bottom line to the first inertial principal axis; *L*1/*L*2, aspect ratio; *M*1/*M*2, diagonal ratio; *I*1/*I*2, the ratio of PMOI of the center of gravity.

**Table 15:** Linear regression index.

Index	*L*1	*L*2	*M*1	*M*2	*I*1	*I*2	*ε*	*β*
*R* ^2^	0.005	0.165	0.001	0.022	0.008	0.035	0.231	0
MAD	0.4919	0.4737	0.4969	0.5034	0.5046	0.5035	0.4909	0.4970

Index	*γ*	*δ*	*θ*	*λ*	*K*	*L*1/*L*2	*M*1/*M*2	*I*1/*I*2
*R* ^2^	0.001	0.032	0.002	0.113	0.208	0.254	0.015	0.092
MAD	0.4964	0.4712	0.4987	0.4890	0.4628	0.4444	0.5017	0.4952

*R*2, goodness of fit; MAD, mean absolute deviation.

**Table 16:** Ranges of ideal form measurement.

Measurement	Range
*S*	641.87∼801.53
*L*2	16.22∼18.28
*K*	–0.21∼2.78
*L*1/*L*2	2.78∼3.15

**Table 17:** Coordinates of characteristic points.

Point	Sample 1		Sample 5	
	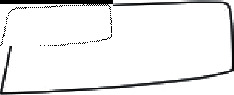		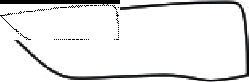	
	*x*	*y*	*x*	*y*
1	–19.71	17.44	–29.20	14.67
2	–11.69	18.09	–12.14	17.09
3	11.43	19.30	5.74	18.83
4	20.49	19.50	16.84	19.34
5	23.52	18.67	20.78	18.37
6	23.52	18.67	20.78	18.37
7	24.14	14.50	21.67	14.34
8	24.46	7.42	21.87	9.52
9	24.32	5.08	21.96	4.29
10	20.89	4.09	17.08	3.27
11	10.50	2.48	12.32	3.25
12	–2.81	1.28	6.15	4.22
13	–12.81	0.65	–2.33	4.01
14	–21.32	0.19	–10.49	3.60
15	–25.08	0	–15.81	3.96
16	–23.56	7.46	–21.13	6.98
17	–22.23	13.17	–25.75	11.23
18	–19.71	17.44	–29.20	14.67

**Table 18:** Evaluation results of product forms.

Form	*S*	*L*2	*K*	*L*1/*L*2
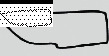	676.33	18.27	6.07	2.81
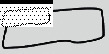	762.81	17.87	–0.52	2.82
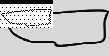	677.50	17.85	5.21	2.96
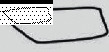	736.39	18.62	0.09	2.88
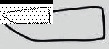	694.25	17.02	–0.10	3.04
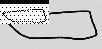	653.56	16.27	–0.15	3.30
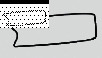	784.86	16.84	0.01	3.16
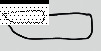	676.88	16.00	–0.18	3.34
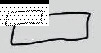	728.07	18.09	–0.53	2.78
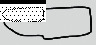	703.17	18.05	5.45	2.78
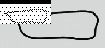	626.48	16.07	–0.17	3.32
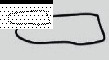	649.21	16.88	–0.40	2.84
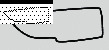	668.41	18.24	5.72	2.80
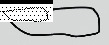	669.70	16.87	–0.40	3.18
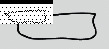	645.40	16.07	–0.17	3.33
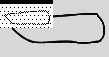	663.43	16.18	–0.17	3.31

**Table 19:** Questionnaire results.

Scheme	1	2
Image value	0.678	0.612

## Data Availability

The data used to support the findings of this study are included within the article.
